# Stellate ganglion block as an intervention in refractory eosinophilic granulomatosis with polyangiitis: a case report

**DOI:** 10.1186/s13223-022-00654-6

**Published:** 2022-02-19

**Authors:** Danxu Ma, Yuting Xue, Rong Shi, Yinan Yang, Huili Li, Xuhua Shi, Li Wang, Yun Wang

**Affiliations:** 1 Department of Anesthesiology and Pain Medicine, Beijing Chaoyang Hospital, Capital Medical University, Beijing, China; 2 Operating Room, Beijing Chaoyang Hospital, Capital Medical University, Beijing, 100020 China; 3 Department of Rheumatology and Immunology, Beijing Chaoyang Hospital, Capital Medical University, Beijing, China; 4 Department of Rheumatology and Immunology, Peking Union Medical College Hospital, Chinese Academy of Medical Sciences, Beijing, China

**Keywords:** Eosinophilic granulomatosis with polyangiitis, EGPA, Stellate ganglion block

## Abstract

**Background:**

Eosinophilic granulomatosis with polyangiitis (EGPA) is a rare vasculitis. Although glucocorticoid therapy with or without immunosuppressants leads to remission in the majority of cases, most EGPA patients remain dependent on glucocorticoid therapy and experience frequent relapses. Here, we report a case of refractory EGPA which responded to stellate ganglion blocks (SGBs).

**Case presentation:**

A 32-year-old woman with aggravated wheezing, purpura, numbness of multiple fingers, and epigastric and abdominal pain was referred to our clinic. Laboratory and radiographic studies led to the diagnosis of EGPA. After an initial favorable response to glucocorticoid and immunosuppressant therapy**,** she experienced a relapse during a glucocorticoid taper. We found that SGB brought symptomatic relief and impeded disease progression. The mechanism of action of SGB on EGPA is undetermined, but may be related to vasodilation, immune modulation, and central nervous system regulation.

**Conclusions:**

This report not only proposes a novel treatment modality for EGPA, but also provides a clinical reference point for further in-depth studies of SGB in multiple immune-linked disorders.

## Background

Eosinophilic granulomatosis with polyangiitis (EGPA), formerly named Churg-Strauss syndrome, is a rare autoimmune disorder characterized by asthma, sinusitis, pulmonary infiltrates, neuropathy, and eosinophilic vasculitis of one or more end-organs. Pathogenesis is thought to involve eosinophilic tissue and vascular infiltration and inflammation induced by a variety of mediators. Although glucocorticoid therapy with or without immunosuppressants leads to remission in the majority of cases, EGPA patients remain dependent on glucocorticoid therapy and experience frequent relapses. Given the side effects of prolonged and high-dose glucocorticoid and immunosuppressive regimens, additional effective therapies are needed [1–4].

Stellate ganglion block (SGB) has been used for the management of sympathetically mediated pain and ischemic disorders of the upper extremity, chest, head and face that include complex regional pain syndrome, postherpetic neuralgia, migraine, tinnitus, and refractory angina [5]. A few case reports have described successful SGB therapy of ischemia and pain caused by vascular complications of rheumatic conditions such as Raynaud disease [6, 7], temporal arteritis [8], and vasculitis induced by systemic lupus erythematosus [9]. Furthermore, SGB has also been used with some success in treating bronchial asthma [10]. Here, we report a case of refractory EGPA with asthma that responded to SGBs.

## Case presentation

**Fig. 1:**
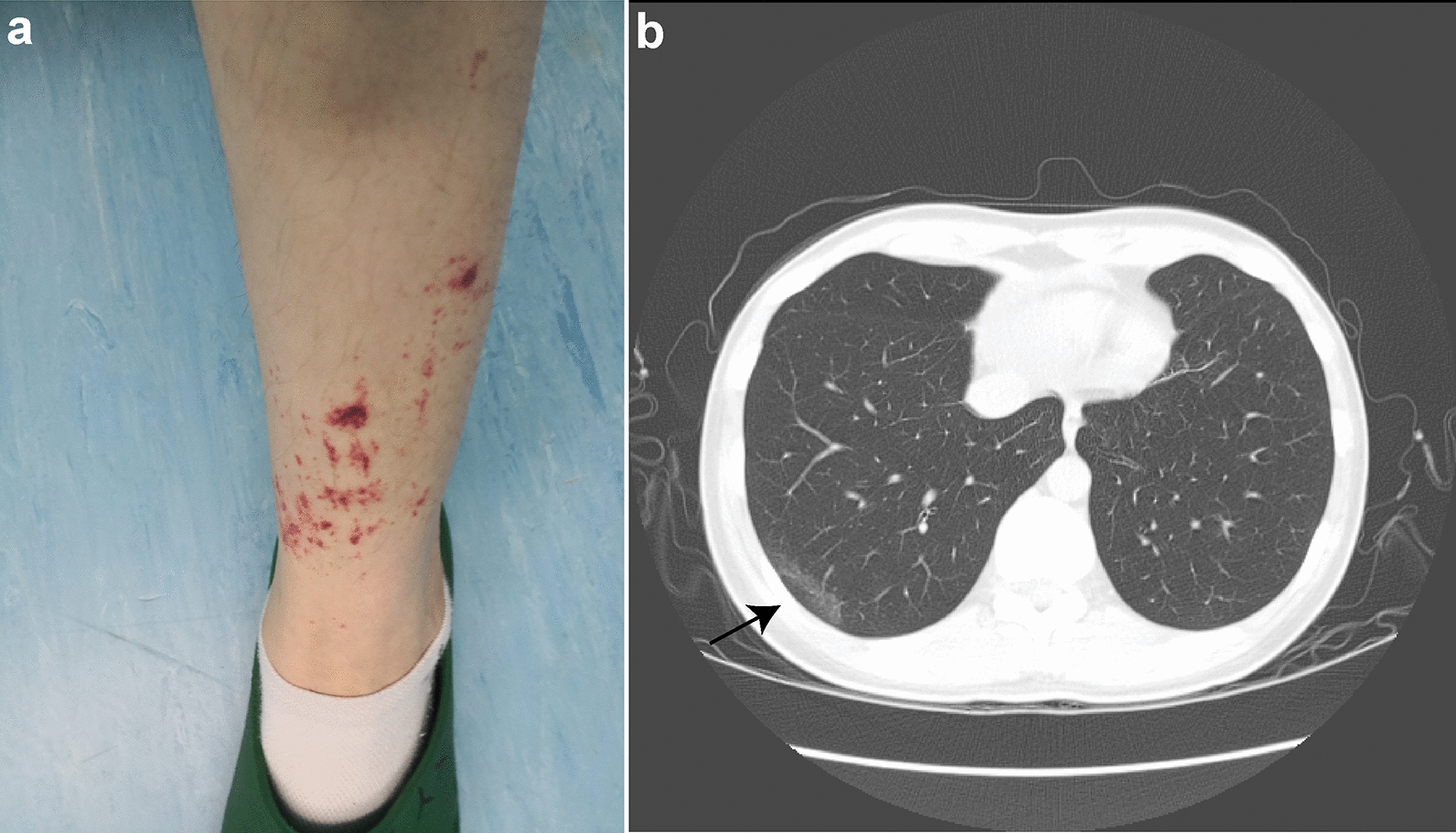
**A** Skin lesion presenting as purpura of the left lower limb. **B** Chest CT scan showed a subpleural patchy shadow in the posterior basal segment of the right lower lobe (black arrow)

A 32-year-old woman complaining of aggravated wheezing, bruising, and numbness of multiple fingers presented to our rheumatology and immunology department. She reported epigastric and right upper abdominal pain of 6 months duration. She had suffered from bronchial asthma for 5 years, treated with inhaled corticosteroid therapy. Physical findings included purpura (Fig. 1a). Laboratory tests revealed increased leukocytosis (15.59 × 10^3^/μL) with eosinophilia (7.96 × 10^3^/μL, 51.1%) and elevated erythrocyte sedimentation rate (ESR) (30 mm/h), C-reactive protein (0.54 mg/dL), immunoglobulin (Ig) E (808 kU/L), IgG4 (2028 mg/L), and rheumatoid factor (176 IU/mL). Negative studies included anti-nuclear antibodies; and cytoplasmic-, perinuclear-, proteinase-3-, and myeloperoxidase-anti-neutrophil cytoplasmic antibodies. Computed tomography of the chest disclosed a subpleural patchy shadow in the posterior basal segment of the right lower lobe (Fig. 1b). She was diagnosed with EGPA according to the 1990 classification criteria of the American College of Rheumatology [4], and began therapy with daily doses of prednisone acetate 30 mg and cyclophosphamide 50 mg given orally. All symptoms resolved quickly, and eosinophilia and ESR returned to normal. Four weeks later, when prednisone acetate was reduced to 27.5 mg, she developed a mild dry nocturnal cough, and experienced a relapse of respiratory, digestive, and neurological symptoms after another 2 weeks, when the daily dose of prednisone acetate was reduced to 25 mg. Consequently, she received supplemental betamethasone 7 mg by intramuscular injection that resulted in a remission of only 1 week’s duration.

**Fig. 2:**
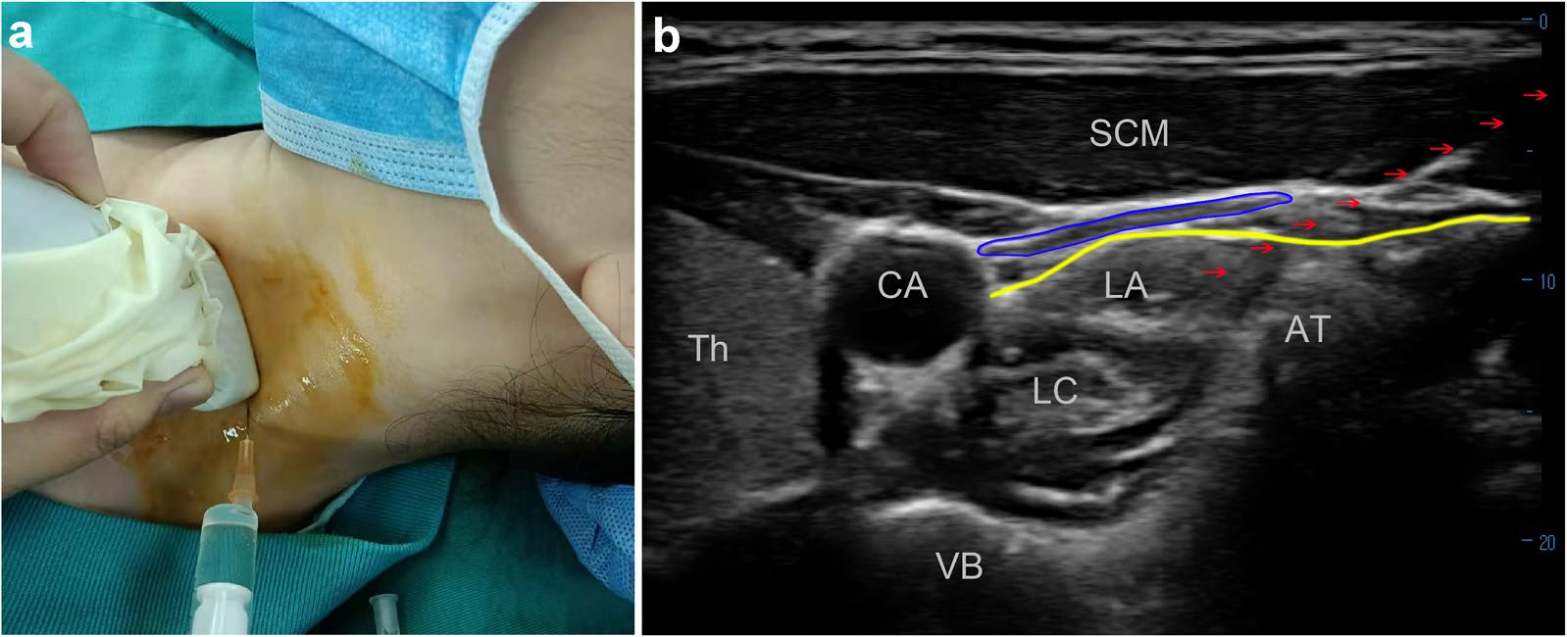
Transducer position and corresponding ultrasound image of the stellate ganglion block (SGB). **A** Performance of the SGB using transverse scanning with needle in-plane approach. **B** Ultrasound images for the SGB. The internal jugular vein was compressed (blue circle); the yellow line represents the prevertebral fascia, and the red arrow indicates the in-plane needle path. *LC* longus colli, *SCM* sternocleidomastoid muscle, *LA* local anesthetic, *Th* thyroid gland, *CA* carotid arter, *AT* anterior tubercle of C6 transverse process, *VB* C6 vertebral body

Considering that SGB is an effective treatment for asthma and multiple ischemic conditions, an experimental ultrasound-guided unilateral SGB was planned. The patient was positioned supine with the neck turned to the right. The skin was disinfected with iodophor, and a high frequency (4–12 MHz) linear-array ultrasound transducer (TUR200, Tuoren Medical Device Co., Ltd. Henan, China) covered with a sterile sleeve was placed transversely at the left anterior cervical region (Fig. 2a) to identify relevant anatomic structures that included the C6 transverse process, carotid artery, internal jugular vein, longus colli muscle, prevertebral fascia, vertebral artery, and inferior thyroid artery. A 25-gauge needle was inserted in-plane and the needle tip was placed between the fascia investing the longus colli muscle and the prevertebral fascia, and 2 mL 1% lidocaine was injected (Fig. 2b).

Immediately after the block, Horner’s syndrome occurred, and she reported simultaneous resolution of all symptoms, such as wheezing, epigastric and abdominal pain, and numbness of the fingers. SGB was repeated every 2 days, alternating between left and right aspects of the neck, for completion of a 7-procedure treatment course. Prednisone acetate was successfully tapered to 22.5 mg daily without symptomatic relapse. Furthermore, no inhaled corticosteroids were required, and improved mood and sleep were reported. Unfortunately, recurrent symptoms were triggered by a negative emotional event, and inhaled corticosteroid therapy was reinstituted to control asthma. A second course of SGBs conferred satisfactory symptomatic relief. The prednisone acetate dose was gradually reduced to 10 mg/day, with cyclophosphamide added at a daily maintenance dose of 50 mg over a 3-month period with no observed recurrence.

## Discussion

Our patient suffered from EGPA involving the respiratory and digestive systems, peripheral nerves, and skin; and experienced a favorable response to glucocorticoid-based induction therapy. However, glucocorticoid dose reduction was difficult, which is in consistent with the observation that EGPA patients frequently relapse during glucocorticoid tapering [4]. To our knowledge, this is the first report of the use of SGB to alleviate EGPA-related symptoms. The mechanism of action is undetermined, and we propose the following possibilities.

Firstly, SGBs may alleviate symptoms and organ damage through vasodilation. The typical pathological feature of EGPA is necrotizing small vessel vasculitis accompanied by eosinophilic infiltrates and perivascular and extravascular granulomas, leading to ischemia of the involved organs [4]. SGB averts sympathetic innervation, resulting in peripheral vasodilatation and increased perfusion [11]; consequently, it is widely used to treat a variety of ischemic disorders of the head, chest, face, and upper extremities. Furthermore, SGB may directly suppress inflammation and edema of the vascular wall [8].

Secondly, SGB significantly impacts conditions linked to immune dysfunction [12]. Though EGPA is considered a classical T helper (Th) 2-response mediated disease, Th1 response cannot be ignored [13]. Eosinophils promote inflammation by releasing cytotoxic granule proteins and lipid mediators [4]. Some Th1-related cytokines are also released during this process, and include interferon-γ, IL-1β, IL-6 and IL-8, which further exacerbate vasculitis and tissue injury [13, 14]. Several studies have demonstrated that SGB led to decreases in concentrations of Th1 cytokines, including IL-1β, IL-6, TNF-α and IL-8 in patients with trauma or chronic ulcerative colitis [15–17]. Therefore, we speculated that SGB could also reduce the tissue damage caused by Th1 cytokines in EGPA. Next, it has been reported that SGB led to reductions of peripheral blood eosinophilia and serum IgE levels in a patient with atopic dermatitis [18], which are also treatment goals of the clinical management of EGPA. Moreover, a recent published hypothesis believes that the effects of the SGB on the immune system are complex and are best represented as immuno-modulating, rather than being simply suppressing or stimulating [12].

Finally, as with other autoimmune diseases [19–22], stress may trigger disease flares in patients with EGPA. Relapse was triggered by a negative emotional event experienced by our patient. SGB has been used with some success to treat multiple psychiatric conditions, including anxiety and post-traumatic stress disorder (PTSD). The mechanism of action in psychiatric conditions is unclear [5, 23], but may involve centripetal neuronal connections between the stellate ganglia and deep brain regions such as the insula, amygdala, and hippocampus, which regulate the formation of cognition, memory, and behaviors [24]. On the other hand, sleep disturbance is common during anxiety and in PTSD patients. SGB may reestablish the normal melatonin circadian rhythm by interrupting the sympathetic cycle [25]. Our patient also reported improvements in mood and sleep after SGBs.

In summary, our case suggests that ultrasound-guided SGB may represent a simple and safe method to relieve symptoms and impede disease progression in EGPA patients, and may lead to wider usage of the technique in immune-linked disorders, such as multiple vasculitides, Sjogren's syndrome, and scleroderma. Further investigation is needed to evaluate efficacy and to elucidate the mechanism of action of SGB in the therapy of immune-linked disorders.

## Data Availability

Data sharing is not applicable to this article as no datasets were generated or analyzed during this case report.

## Abbreviations

EGPA: Eosinophilic granulomatosis with polyangiitis
SGB: Stellate ganglion block
PTSD: Post-traumatic stress disorder
